# Granulocyte macrophage colony-stimulating factor-induced macrophages of individuals with autism spectrum disorder adversely affect neuronal dendrites through the secretion of pro-inflammatory cytokines

**DOI:** 10.1186/s13229-024-00589-2

**Published:** 2024-02-21

**Authors:** Ryohei Takada, Michihiro Toritsuka, Takahira Yamauchi, Rio Ishida, Yoshinori Kayashima, Yuki Nishi, Mitsuru Ishikawa, Kazuhiko Yamamuro, Minobu Ikehara, Takashi Komori, Yuki Noriyama, Kohei Kamikawa, Yasuhiko Saito, Hideyuki Okano, Manabu Makinodan

**Affiliations:** 1 Department of Psychiatry, Nara Medical University School of Medicine, 840 Shijo-Cho, Kashihara City, Nara, 634-8522 Japan; 2 Department of Physiology, Keio University School of Medicine, 35 Shinanomachi, Shinjuku-Ku, Tokyo, 160-8582 Japan; 3 Department of Neurophysiology, Nara Medical University School of Medicine, 840 Shijo-Cho, Kashihara City, Nara, 634-8522 Japan; 4 Osaka Psychiatric Research Center, 3-16-21 Miyanosaka, Hirakata City, Osaka 573-0022, Japan

**Keywords:** Autism spectrum disorder, Human iPS cell, Macrophage, Interleukin-1α, Tumor necrosis factor-α, Dendrite

## Abstract

**Background:**

A growing body of evidence suggests that immune dysfunction and inflammation in the peripheral tissues as well as the central nervous system are associated with the neurodevelopmental deficits observed in autism spectrum disorder (ASD). Elevated expression of pro-inflammatory cytokines in the plasma, serum, and peripheral blood mononuclear cells of ASD has been reported. These cytokine expression levels are associated with the severity of behavioral impairments and symptoms in ASD. In a prior study, our group reported that tumor necrosis factor-α (TNF-α) expression in granulocyte–macrophage colony-stimulating factor-induced macrophages (GM-CSF MΦ) and the TNF-α expression ratio in GM-CSF MΦ/M-CSF MΦ (macrophage colony-stimulating factor-induced macrophages) was markedly higher in individuals with ASD than in typically developed (TD) individuals. However, the mechanisms of how the macrophages and the highly expressed cytokines affect neurons remain to be addressed.

**Methods:**

To elucidate the effect of macrophages on human neurons, we used a co-culture system of control human-induced pluripotent stem cell-derived neurons and differentiated macrophages obtained from the peripheral blood mononuclear cells of five TD individuals and five individuals with ASD. All participants were male and ethnically Japanese.

**Results:**

Our results of co-culture experiments showed that GM-CSF MΦ affect the dendritic outgrowth of neurons through the secretion of pro-inflammatory cytokines, interleukin-1α and TNF-α. Macrophages derived from individuals with ASD exerted more severe effects than those derived from TD individuals.

**Limitations:**

The main limitations of our study were the small sample size with a gender bias toward males, the use of artificially polarized macrophages, and the inability to directly observe the interaction between neurons and macrophages from the same individuals.

**Conclusions:**

Our co-culture system revealed the non-cell autonomous adverse effects of GM-CSF MΦ in individuals with ASD on neurons, mediated by interleukin-1α and TNF-α. These results may support the immune dysfunction hypothesis of ASD, providing new insights into its pathology.

**Supplementary Information:**

The online version contains supplementary material available at 10.1186/s13229-024-00589-2.

## Background

Autism spectrum disorder (ASD) is a complex neurodevelopmental disorder characterized by impaired social interaction, poor communication skills, and restricted or repetitive patterns of behavior and interests [1]. The worldwide prevalence of autism has steadily increased in recent decades, which is still about 1% [2, 3]. Although its etiology and pathogenesis have not been fully elucidated, a growing body of evidence suggests that immune dysfunction and inflammation are associated with the neurodevelopmental deficits observed in ASD [4–7]. For example, a significant association between common allergic conditions (food, respiratory, or skin allergy) and autoimmune diseases in ASD has been reported [8, 9], suggesting that there are systemic abnormalities of the immune system both in the periphery and in the central nervous system (CNS) of patients with ASD. Postmortem studies have revealed the existence of neuroinflammation in the CNS, as indicated by the expression of the genes of immune-related signaling pathways, microglial activation, and upregulation and mutation of microglia-related genes in the brains of patients with ASD [10–12]. In the periphery, pro-inflammatory cytokines were elevated in the plasma, serum, and peripheral blood mononuclear cells (PBMCs) of individuals with ASD, which was associated with the severity of behavioral impairments and associated with symptoms in children with ASD [13, 14]. Epigenetic changes in hematopoietic cells during embryogenesis reportedly alter definitive hematopoiesis and microglial development, leading to such immune abnormalities [15].

It has been believed that the CNS is protected from the periphery by the blood–brain barrier (BBB). Depending on their type, cytokines can pass through the BBB [16], whereas peripheral immune cells face challenges in accessing the brain; however, recent reports have shown that the integrity of the BBB may be disrupted by the systemic inflammation observed in ASD [17, 18]. In addition, even without disruptive BBB changes, peripheral cytokines and peripheral immune cells, such as monocytes and macrophages, can be secreted or activated by social stress and systemic inflammation. These cytokines and immune cells may cause the activation of endothelial cells and perivascular macrophages, which release secondary messengers to affect the brain [19–22].

Macrophages are diverse, highly plastic, mononuclear phagocytic cells with a functional similarity to microglia, which play critical roles in CNS health and disease. Although the origin of macrophages, especially monocyte-derived intravascular macrophages, is known to be different from microglia [23], microglia are nevertheless considered tissue-resident macrophages. Additionally, a recent report identified another type of macrophage associated with the CNS, which is distinct from both microglia and intravascular macrophages, named CNS-associated macrophages (CAMs) [24]. CAMs can be divided into two types of cells; one of these, termed perivascular macrophages, settles in the perivascular space in the brain soon after birth and has the potential to interact with intravascular macrophages. This interaction with macrophages could affect brain cells, including microglia and neurons [24].

Microglia and macrophages exhibit a spectrum of phenotypes [25, 26]. We have reported that the expression of tumor necrosis factor-α (TNF-α), a pro-inflammatory cytokine, in granulocyte–macrophage colony-stimulating factor-induced macrophages (GM-CSF MΦ; classic nomenclature “M1 MΦ”), and the TNF-α expression ratio in GM-CSF MΦ/M-CSF MΦ (macrophage colony-stimulating factor-induced macrophages; classic nomenclature “M2 MΦ”) are markedly higher in individuals with ASD than in typically developed (TD) individuals [27]. Therefore, we focused our investigation on how peripheral macrophages affect brain cells in individuals with ASD. In rodent models, repeated environmental stress induces the activation of microglia in the medial prefrontal cortex (mPFC) to express pro-inflammatory cytokines such as interleukin-1α (IL-1α) and TNF-α, which leads to the shortening of and reduced branching of neural dendrites and leads to social avoidance behavior [28, 29]. This mechanism appears relevant to ASD, as those with autistic traits are prone to stress and anxiety, resulting in a higher prevalence of social withdrawal [30, 31]. We previously reported that impaired social interaction during childhood is related to elevated blood levels of TNF-α in individuals with ASD [32]; thus, the highly expressed pro-inflammatory cytokines in macrophages of individuals with ASD [27] may have a similar effect on brain morphology and behavior.

Herein, we hypothesized that macrophages in individuals with ASD would affect neuronal cells differentially from that of TD individuals due to their inflammatory phenotype. To investigate this hypothesis, we co-cultured human-induced pluripotent stem cell (hiPSC)-derived neurons with differentiated GM-CSF MΦ or M-CSF MΦ from TD individuals and individuals with ASD. We examined how macrophages cause morphological changes in neurons.

## Methods

### Participants and clinical assessments

Five individuals with ASD (age: 31.0 ± 7.25 years) and five TD individuals (age: 34.8 ± 7.60 years) of Japanese ethnicity were enrolled. All participants were male and born and living in Japan. The participation criteria and assessment of clinical features were the same as those described previously [27]. Briefly, individuals with ASD were recruited from the outpatient service of the Department of Psychiatry at Nara Medical University Hospital. ASD diagnosis was based on the Diagnostic and Statistical Manual of Mental Disorders, Fifth Edition criteria. At least two experienced psychiatrists examined each individual separately, and a diagnostic consensus was reached. Further evaluation was performed using the Autism Diagnostic Observation Schedule-2 (ADOS-2) by psychiatrists and trained staff [33]. Autism symptom severity was assessed via self-reporting using the Autism Quotient-Japanese version (AQ-J) [34, 35]. All participants had average intelligence higher than the full intelligence quotient (FIQ) of 70, estimated using the Similarities and Symbol search subtests of the Wechsler Adult Intelligence Scale, 3rd ed. [36]. Participants had no other neurological disorders, mental illnesses, infectious diseases, autoimmune diseases, or steroid use. This study was approved by the appropriate ethics committees of Nara Medical University and was conducted as per the Code of Ethics of the World Medical Association (Declaration of Helsinki) for experiments involving humans. All participants were given a complete description of the study and provided written informed consent before enrollment.

### Neuronal differentiation of hiPSCs

Two human iPSC lines, 201B7 [37] and 1008C15, were used as healthy control hiPSC lines. The 1008C15 line was established from human dermal fibroblasts of a TD male based on a previously described method in our laboratory [38]. Human iPSCs were cultured in StemFit AK02N (Ajinomoto, Tokyo, Japan), plated onto a 35 mm culture dish coated with iMatrix-511 silk (Nippi, Tokyo, Japan) without feeder cells at 37 °C in humidified air containing 5% CO2.

Neuronal differentiation was performed according to the previously described method [39]. Briefly, the following plasmid vectors were used to establish *NEUROG2*-inducible hiPSCs: PB-TET-PH-lox66FRT-*NEUROG2*, pCMV-HyPBase-PGK-Puro, and PB-CAGrtTA3G-IH. These vectors were co-transfected into hiPSCs using Gene Juice Transfection Reagent (Novagen, Madison, Wisconsin, USA). Transfectants were cultured in StemFit AK02N containing 150 μg/ml hygromycin (Wako Pure Chemical Industries, Ltd., Osaka, Japan) and 0.1–1.0 μg/ml puromycin (Sigma-Aldrich, St. Louis, MO, USA). To induce glutamatergic neurons, these *NEUROG2*-inducible hiPSCs were dissociated and seeded on poly-ornithine (Sigma-Aldrich) and iMatrix-511 silk-coated coverslips in 24-well plates at 5 × 10^4^ cells/well, and cultured in neural induction medium; Neurobasal Plus medium supplemented with B-27, 1 × Glutamax (Invitrogen, Thermo Fisher Scientific Inc., Waltham, MA, USA), 20 μM Y-27632 (Wako Pure Chemical Industries, Ltd.), 20 μM DAPT (Sigma-Aldrich), 100 μg/ml G418 (Nacalai Tesque, Kyoto, Japan), and 1 μg/ml doxycycline (Wako Pure Chemical Industries, Ltd.). After five days, the medium was replaced with neuron culture medium: Neurobasal Plus medium supplemented with B-27, 1 × Glutamax, 10 ng/ml brain-derived neurotrophic factor (BDNF; R&D Systems, Minneapolis, MN, USA), 10 ng/ml glial cell line-derived neurotrophic factor (GDNF; Alomone-Labs, Jerusalem, Israel), 200 μM L-ascorbic acid (Sigma-Aldrich), 200 μM dibutyryl cyclic adenosine monophosphate sodium salt (Nacalai Tesque), 10 ng/ml neurotrophin-3 (Alomone-Labs), 1 × CultureOne Supplement (GIBCO, Thermo Fisher Scientific Inc.), and 1% penicillin–streptomycin mixed solution (Nacalai Tesque). The medium was half-replaced twice a week for up to 56 days.

### Monocyte isolation and macrophage differentiation

Monocyte isolation was performed with a magnetic-activated cell sorting system (Miltenyi Biotec, Bergisch Gladbach, Germany) and CellXVivo Human M1 or M2 Macrophage Differentiation Kit (R&D Systems), according to the manufacturer’s protocol. Briefly, whole human blood samples were collected via venipuncture, and PBMCs were separated by density-gradient centrifugation using the separation medium, Lymphoprep (Axis Shield, Oslo, Norway), and separation tubes, Leucosep (Greiner Bio-One, Kremsmünster, Austria). CD14 + monocytes were isolated from PBMCs using a magnetic-activated cell sorting system with CD14 microbeads (Miltenyi Biotec). For macrophage differentiation, CD14 + monocytes were resuspended in a phosphate-buffered saline solution (PBS) (Wako Pure Chemical Industries, Ltd) containing 0.5% bovine serum albumin (Sigma-Aldrich), 2 mM ethylenediaminetetraacetic acid, and 1% penicillin–streptomycin mixed solution (Nacalai Tesque). Cells were seeded on 12-well plates coated with poly-L-lysine (IWAKI, Shizuoka, Japan) at a density of 1 × 10^6^ cells/ml and cultured in differentiation medium containing recombinant human granulocyte–macrophage colony-stimulating factor or recombinant human macrophage colony-stimulating factor, at 37 °C in humidified air containing 5% CO2. On day 3, half of the culture medium was replaced with fresh medium, and GM-CSF MΦ or M-CSF MΦ were collected on day 6 using a cell scraper for co-culture with neurons and subsequent analysis. Some collected macrophages were seeded in 24-well plates coated with poly-L-lysine at a density of 5 × 10^5^ cells/well and cultured in neuron culture medium for up to 28 days for quantitative reverse transcription-polymerase chain reaction (qRT-PCR) analysis. Macrophage differentiation for participants 1–3 in the TD group and 1–3 in the ASD group was performed twice, and only once for participants 4 and 5 of both groups.

### Co-culture of neurons and macrophages

Glutamatergic neurons prepared as described above were used on day in vitro 28 (DIV28). For a direct co-culture, differentiated macrophages were collected and reseeded on cultured neurons in a neuron culture medium at a density of 5 × 10^4^ cells/well. For indirect co-culture, macrophages were reseeded on a culture insert (Falcon, NY, USA) coated with iMatrix-511 silk at 5 × 10^4^ cells per insert in neuron culture medium, and the macrophage-containing culture inserts were suspended in 24-well plates. The medium was half-replaced twice per week for an additional 28 days. For each macrophage differentiation, one differentiation of hiPSC-derived neurons was performed; TDs and ASDs were collected in pairs to minimize errors due to neuronal differentiation.

### Addition of cytokines and neutralizing antibodies

To examine the cytokine effect, the neuron culture medium was replaced with either or both recombinant human TNF-α and IL-1α proteins (Peprotech, Cranbury, NJ, USA) at a concentration of 10–100 ng/ml at DIV28. The cytokine-containing medium was half-replaced twice a week for an additional 28 days.

For the neutralizing antibody administration experiment, the neuron culture medium was replaced with either or both neutralizing antibodies of anti-human TNF-α (BioLegend, San Diego, CA, USA) and anti-human IL-1α (InvivoGen, San Diego, CA, USA) at a concentration of 1 μg/ml at the start of macrophage co-culture. For the negative control, either or both antibodies were replaced with the same amount of corresponding IgG isotype antibodies. The antibody-containing medium was half-replaced twice a week for an additional 28 days.

### Immunocytochemistry (ICC)

Cells were fixed in PBS containing 4% paraformaldehyde for 30 min at room temperature, and then rinsed twice with PBS. Thereafter, cells were blocked and permeabilized for 1 h in blocking buffer (PBS containing 5% donkey- or goat-serum albumin and 0.3% Triton-X) and incubated with the primary antibodies diluted in blocking buffer at 4 ℃ overnight. After washing thrice with PBS, cells were incubated with Alexa Fluor 488-, 546-, or 633-conjugated secondary antibodies (Invitrogen) at room temperature for 1 h. Nuclei were stained using 4´6-diamidino-2-phenyl-indole, dihydrochloride (DAPI; Invitrogen) in PBS, rinsing the cells after incubation with secondary antibodies.

Images were randomly acquired using a Nikon C2 confocal laser microscope with the NIS-Elements AR software (Nikon, Tokyo, Japan). For dendrite analysis, two to three images were collected from each coverslip, and images were randomly acquired across fields using a 20 × objective lens. Images were taken as z-stack images with a 1 μm interval for five stacks. Merged images of the stacks were used for analysis. Microtubule-associated protein 2 (MAP2)-positive cells were identified for each image, and the length of MAP2-positive dendrites extending from the cell body was measured. MAP2-positive dendrites were traced and measured manually in a blinded fashion by one researcher using the NIH Image J software. The average, total, and most extended dendrite lengths and total branch numbers of dendrites were quantified (Additional file 1: Fig. S1A). Overlapping dendrites were distinguished from which cell they were from upon reviewing the unmerged z-stack images. Cells with MAP2-positive dendrites that did not fit in the image were excluded from the analysis. Each parameter of MAP2-positive cells was averaged for each image to create a single data point. The number of MAP2-positive cells was calculated by counting the number of both MAP2 and DAPI immunopositive cells in the image.

### Statistical analysis

Differences in demographic characteristics (age, AQ-J score, and FIQ) between individuals with ASD and TD individuals were examined using an unpaired t test. Comparisons of MAP2 + dendrite data were performed by one-way analysis of variance (ANOVA) with post hoc multiple comparison test using Tukey’s honest significant test (HSD) or Dunnett’s multiple comparison test in the study of cytokine addition compared with the vehicle group. When the standard deviations were shown to be significantly different by Bartlett’s test, the Brown–Forsythe ANOVA test and Dunnett’s T3 multiple comparison tests were used. When comparing two groups, an unpaired t test or Welch’s t test was used. Data are presented as medians with interquartile ranges.

All statistical analyses were performed using Prism 9 (GraphPad, Inc., La Jolla, CA, USA), and differences were considered significant at p < 0.05.

## Results

Table 1 presents the participants’ demographic data. There were no significant differences in age (t = 0.809, p = 0.442) or FIQ score (t = 0.350, p = 0.735) between the ASD and TD groups. As expected, individuals with ASD had significantly higher AQ-J scores than TD individuals (t = 5.33, p = 0.0007).

**Table 1:** Demographic data of the study participants

	TD	ASD	
	(n = 5)	(n = 5)	
Age (years)	34.8 (7.60)	31.0 (7.25)	t = 0.809, p = 0.442
FIQ	110 (9.14)	108 (12.1)	t = 0.350, p = 0.735
AQ-J	14.0 (6.96)	34.2 (4.82)	t = 5.33, p = 0.0007

Data presented as mean (SD), and unpaired t test was performed

### Neuronal differentiation of hiPSCs into glutamatergic excitatory neurons and co-culture with macrophages

**Fig. 1:**
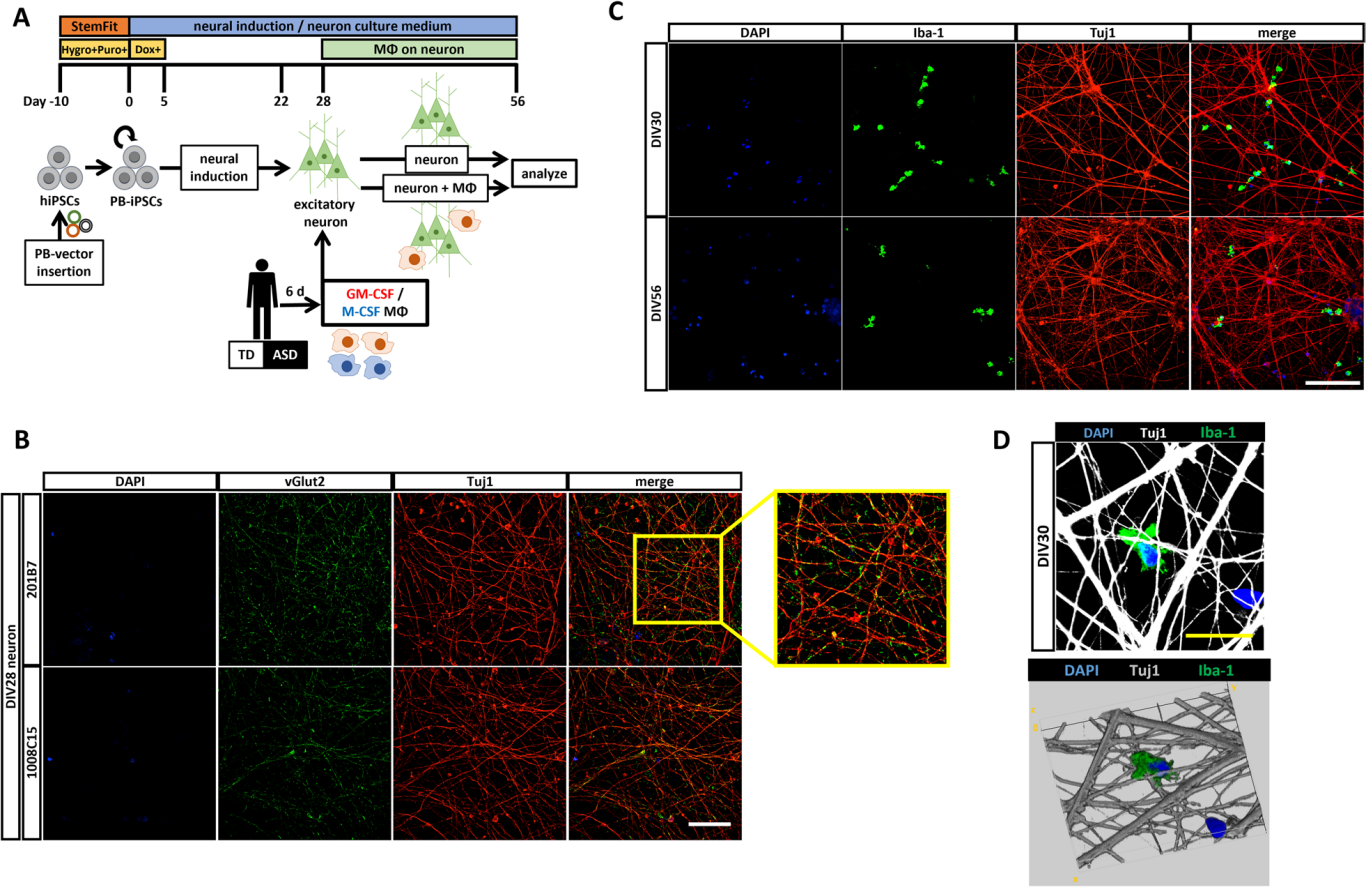
Co-culture system of hiPSC-derived neurons and macrophages. **A** Summary of neuronal differentiation of hiPSCs into glutamatergic neurons and the macrophage differentiation and co-culture protocol. **B** Immunocytochemistry at DIV28 to characterize hiPSCs-derived glutamatergic neurons of two hiPS cell lines. Expression of excitatory neuronal marker vesicular glutamate transporter 2 (vGlut2) on neuron-specific class III beta-tubulin (Tuj-1) observed. Scale bar: 30 μm. **C** Immunocytochemistry at DIV30, 2 days after co-culture, and DIV56. Ionized calcium-binding adapter molecular 1 (Iba1) immunopositive macrophages of TD1 were observed on hiPSC-derived neurons from 201B7 hiPSC line. Scale bar: 100 μm. **D** 3D-reconstitution of the upper image by NIH ImageJ volume viewer shown below. The upper image was reconstructed and rotated 175 degrees on the x-axis, 40 degrees on the y-axis, and 75 degrees on the z-axis. Scale bar: 20 μm

For neuronal differentiation, we prepared two hiPSC lines from healthy controls (Additional file 1: Fig. S1B). The protocol for the baseline analysis using the co-culture system is shown in Fig. 1A. A previous study [39] showed that hiPSC-derived neurons induced by the same method in this study were almost all glutamatergic excitatory neurons. This was confirmed by examining the expression of beta III tubulin (Tuj-1) and vesicular glutamate transporter 2 (vGlut2) at DIV28 by ICC (Fig. 1B), and the absence of glial fibrillary acidic protein (GFAP)-positive astrocytes at DIV56 was also confirmed by ICC (Additional file 1: Fig. S1C). In addition, we evaluated the electrophysiological features of hiPSC-derived neurons using the whole-cell patch-clamp method at DIV28. Action potentials and spontaneous excitatory postsynaptic currents were recorded from both hiPSC line-derived neurons, indicating the neuronal viability and functional connectivity (Additional file 1: Fig. S1D). We further confirmed that ionized calcium-binding adapter molecule 1 (Iba1) immunopositive macrophages were present on neurons using ICC at DIV30, two days after the start of co-culture, and at DIV56 (Fig. 1C and D). The polarization of differentiated macrophages was verified by qRT-PCR at day 6 in macrophage differentiation medium (Additional file 1: Fig. S1E). GM-CSF MΦ showed an inflammatory profile of high expression of *IL-1α* and low expression of *IL-10*, while M-CSF MΦ showed an anti-inflammatory profile with opposite expression pattern in both TD and ASD groups.

### ASD-GM-CSF MΦ induce significantly greater shortening of dendrites than TD-GM-CSF MΦ

**Fig. 2:**
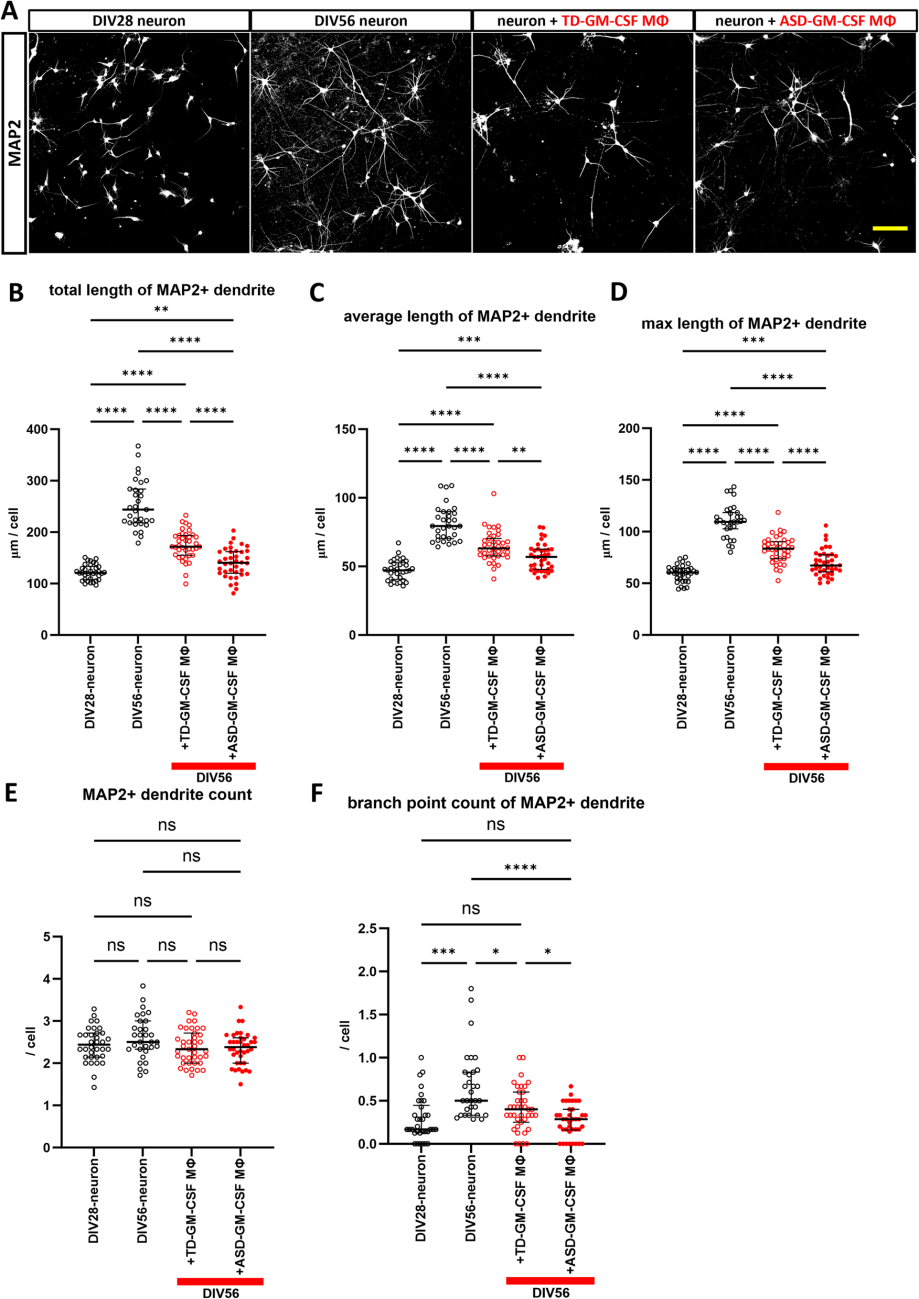
Dendritic changes induced by GM-CSF MΦ. **A** Representative images of immunostaining of MAP2 + dendrites. All neurons were induced from 201B7 hiPSC line, and neuron only or co-cultured with macrophages of TD2 or ASD3 were used in these images. Scale bar: 100 μm. **B**–**F** Results of (**B**) total length of MAP2 + dendrite, **C** average length of MAP2 + dendrite, **D** max length of MAP2 + dendrite, **E** MAP2 + dendrite count, and **F** branch point count of MAP2 + dendrite. Co-culture of GM-CSF MΦ-induced shortening of dendrites compared to culturing only neurons at DIV56, and ASD-GM-CSF MΦ exerted more severe effects than TD-GM-CSF MΦ. **B** Brown–Forsythe ANOVA test, F (3.00, 78.20) = 107.6, p < 0.0001, with post hoc multiple comparison test of Dunnett’s T3. **C** Brown–Forsythe ANOVA test, F (3.00, 115.5) = 62.56, p < 0.0001, with post hoc multiple comparison test of Dunnett’s T3. **D** Brown–Forsythe ANOVA test, F (3.00, 109.1) = 98.52, p < 0.0001, with post hoc multiple comparison test of Dunnett’s T3. **E** One-way ANOVA test, F (3, 138) = 2.426, p = 0.0682, with post hoc Tukey’s multiple comparison test. **F** Brown–Forsythe ANOVA test, F (3.00, 93.22) = 14.30, p < 0.0001, with post hoc multiple comparison test of Dunnett’s T3. n(DIV28-neuron) = 34 fields of 16 independent dishes from five times differentiations of two control healthy hiPSC lines each, n(DIV56-neuron) = 31 fields of 14 independent dishes from five times (201B7) and 4 times (1008C15) differentiations of two control healthy hiPSC lines each, n(TD-GM-CSF MΦ) = 39 fields of 19 independent dishes from five times differentiations of two control healthy hiPSC lines each, and n(ASD-GM-CSF MΦ) = 38 fields of 18 independent dishes from five times differentiations of two control healthy hiPSC lines each. *p < 0.05, **p < 0.01, ***p < 0.001, ****p < 0.0001

To evaluate the effect of macrophages on neurons during co-culture, we investigated the morphology of MAP2 immunopositive dendrites by measuring their length and number. DIV56-neuron showed a more mature phenotype compared to DIV28-neuron with longer dendrite length and many dendritic branches, but the number of dendrites showed no difference (Fig. 2A–F and Additional file 1: Fig. S2A). This result suggests that basic cell morphology, including dendrite projection, is nearly completed by DIV28, with maturation and further branching progressing by DIV56 in this co-culture system. However, GM-CSF MΦ, in both TD and ASD, may inhibit this maturation process. Neurons cultured with GM-CSF MΦ showed shorter lengths and less branches than neurons cultured without GM-CSF MΦ at DIV56, and this inhibitory effect was severe in ASD-GM-CSF MΦ with a significant difference compared to TD-GM-CSF MΦ (Fig. 2A–F). There was no significant difference in the number of MAP2 + neurons between DIV56-neuron and neurons co-cultured with GM-CSF MΦ. Therefore, it is unlikely that these dendritic changes were caused by the death of MAP2 immunopositive neurons (Additional file 1: Fig. S2B). Comparing neurons cultured with GM-CSF MΦ to DIV28-neuron, the dendrite length was significantly shorter in DIV28-neuron (Fig. 2B–D). Still, the number of branch points (Fig. 2F) and the number of dendrites (Fig. 2E) remained the same in all groups. There was no significant difference in MAP2 + dendrites between neurons cultured with M-CSF MΦ, and their length, number, and branch points were comparable to DIV56-neuron (Additional file 1: Fig. S2C–H).

### GM-CSF MΦ induce dendritic shortening by humoral factor secretion

**Fig. 3:**
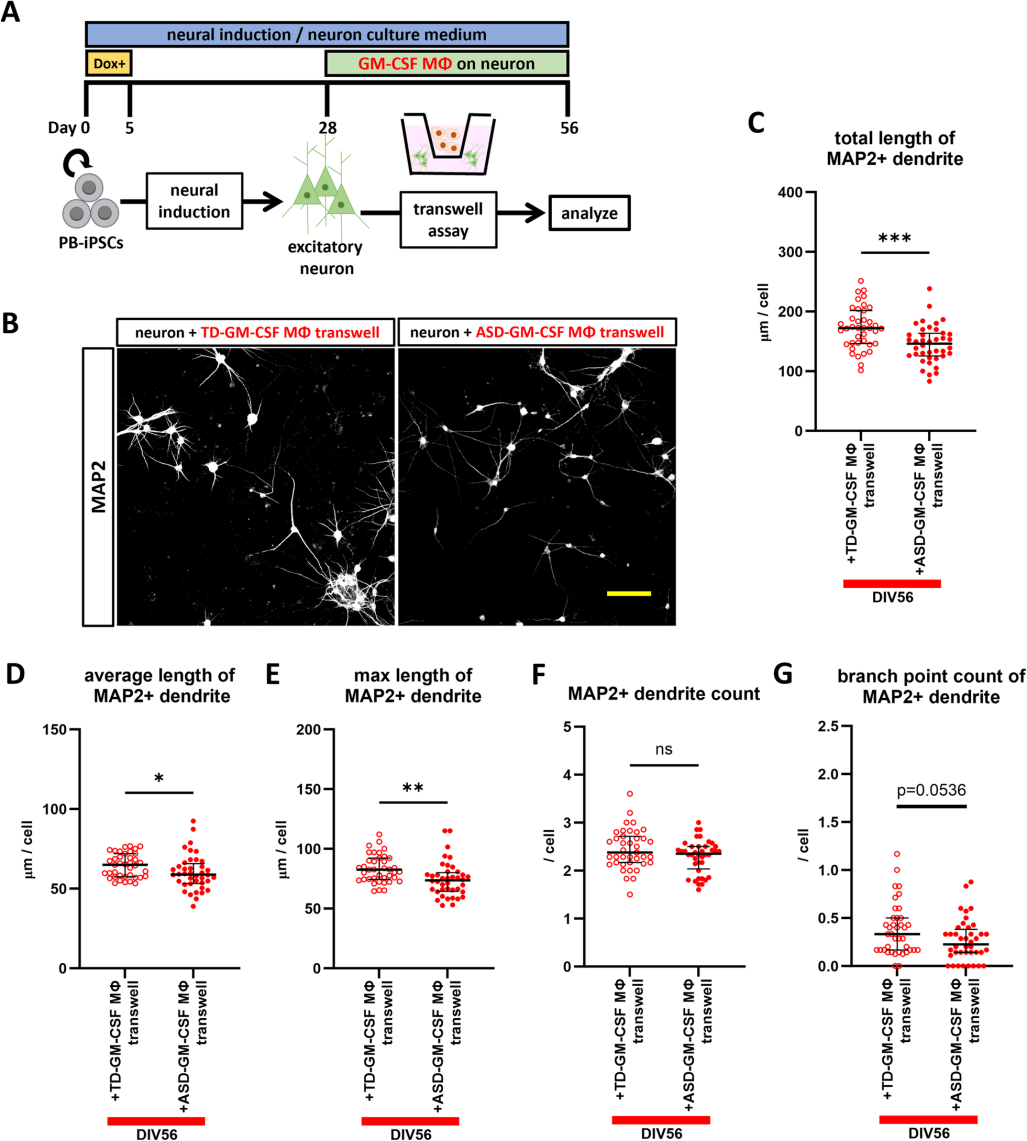
Transwell assay of GM-CSF MΦ. **A** Summary of the transwell assay. **B** Representative images of immunostaining of MAP2 + dendrite cultured with TD- or ASD-GM-CSF MΦ indirectly at DIV56. All neurons were induced from 201B7 hiPSC line, and co-cultured with macrophages of TD3 or ASD1 were used in these images. Scale bar: 100 μm. **C**–**G** Results of (**C**) total length of MAP2 + dendrite, **D** average length of MAP2 + dendrite, **E** max length of MAP2 + dendrite, **F** MAP2 + dendrite count, and **G** branch point count of MAP2 + dendrite. Similar dendritic shortening to the results of direct co-culture was observed in transwell assay. **C** Unpaired t test, t = 3.592, p = 0.0006. **D** Welch’s t test, t = 2.010, p = 0.0484. **E** Unpaired t test, t = 2.956, p = 0.0041. **F** Unpaired t test, t = 1.757, p = 0.0830. **G** Unpaired t test, t = 1.960, p = 0.0536. n(TD-GM-CSF MΦ transwell) = 39 fields of 19 independent dishes from five times differentiations of two control healthy hiPSC lines each, and n(ASD-GM-CSF MΦ transwell) = 40 fields of 20 independent dishes from five times differentiations of two control healthy hiPSC lines each. *p < 0.05, **p < 0.01, ***p < 0.001

We performed a transwell assay to elucidate whether the GM-CSF MΦ-induced dendritic shortening is contact-dependent or not (Fig. 3A). The results were similar to those of the direct-contact co-culture with GM-CSF MΦ, with comparable data except for the number of branches that remained in a significant trend between TD-GM-CSF MΦ and ASD-GM-CSF MΦ (Fig. 3B–G). Therefore, we hypothesized that humoral factors secreted by GM-CSF MΦ affect dendrites. Among such humoral factors, we focused on pro-inflammatory cytokines because, as mentioned above, a previous study reported that both TNF-α and IL-1α induce dendritic atrophy in mouse models [29], and a human study has also reported that a mixture of inflammatory cytokines (TNF-α, IL-1β, IL-6, IL-17A, and interferon-γ (IFN-γ)) decrease the neurite outgrowth of human neural precursor cell line-derived neurons [40]. Since IFN-γ has been reported to increase neurite outgrowth in a hiPSC model [41], gene expression of *IFN-γ* was not specific to GM-CSF MΦ from our qRT-PCR analysis (Additional file 1: Fig. S1E), and we reported higher gene expression of *TNF-α* and no difference in *IL-6* and *IL-17RA* expression in GM-CSF MΦ of individuals with ASD in our previous study [27], and we focused on TNF-α and IL-1α in this study. However, our previous data were obtained from macrophages soon after differentiation from PBMCs for 6 days in macrophage differentiation medium; thus, we analyzed the gene expression of GM-CSF MΦ after 28 days of culture in neuron medium to mimic the co-culture condition (Additional file 1: Fig. S3A). Quantitative RT-PCR analysis showed a higher expression of *IL-1α* in ASD-GM-CSF MΦ than in TD-GM-CSF MΦ, but the expression level of *TNF-α* showed only a tendency to increase, which is consistent with the trend from the results of a previous study (Additional file 1: Fig. S3B and C).

### Pro-inflammatory cytokines have the potential to shorten the dendrites of hiPSC-derived excitatory neurons

**Fig. 4:**
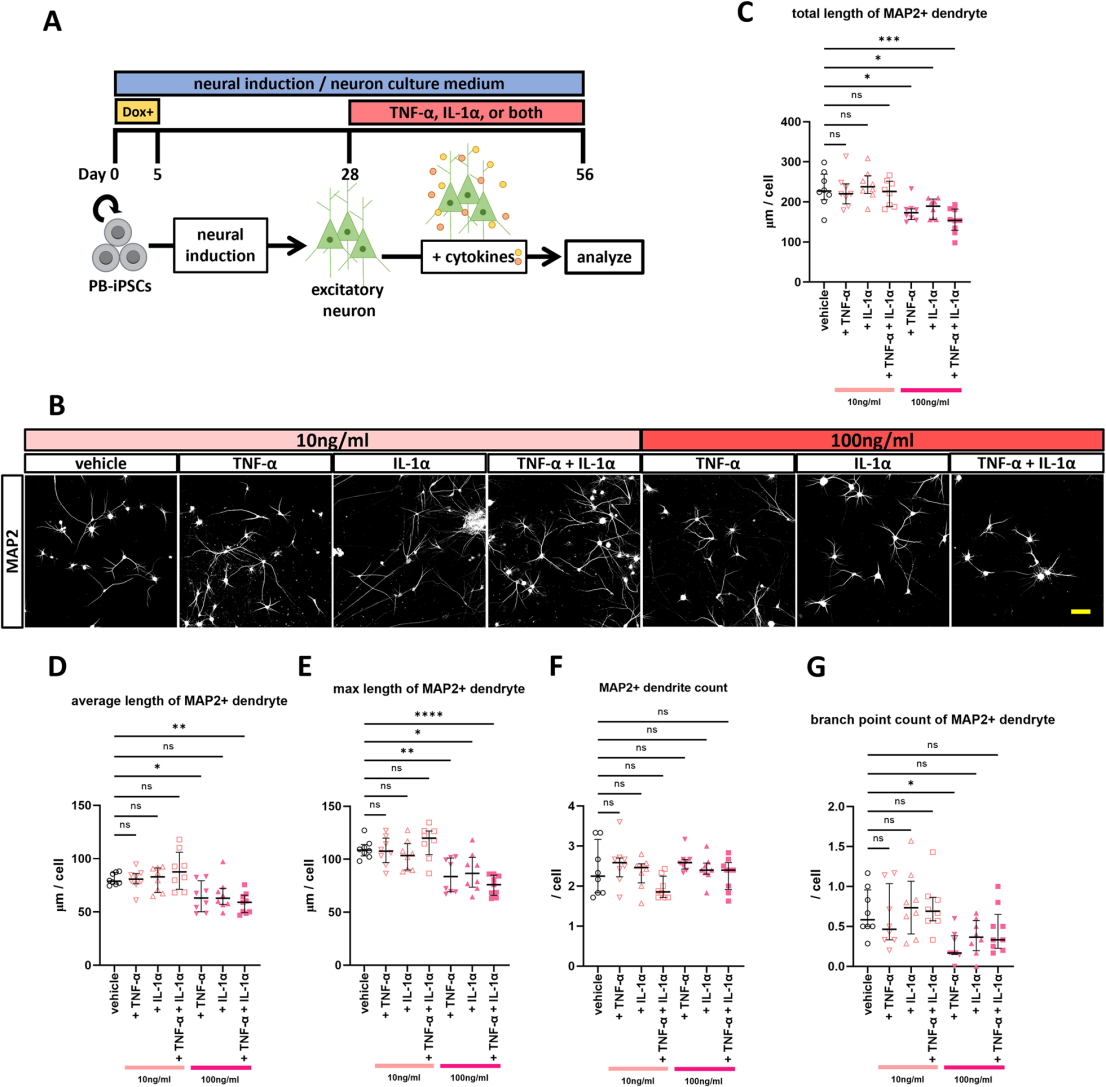
Effects of pro-inflammatory cytokines on neuronal dendrites. **A** Summary of the cytokine addition assay. **B** Representative images of immunostaining of MAP2 + dendrites cultured with TNF-α, IL-1α, or both at DIV56. All neurons induced from 201B7 hiPSC line used in these images. Scale bar: 100 μm. **C**–**G** Results of (**C**) total length of MAP2 + dendrite, **D** average length of MAP2 + dendrite, **E** max length of MAP2 + dendrite, **F** MAP2 + dendrite count, and **G** branch point count of MAP2 + dendrite. Addition of pro-inflammatory cytokines in the culture medium-induced shortening of the total length of MAP2 + dendrites in a manner similar to co-culture with GM-CSF MΦ. **C** One-way ANOVA test, F (6, 50) = 8.107, p < 0.0001, with post hoc Dunnett’s multiple comparison test. **D** One-way ANOVA test, F (6, 50) = 6.918, p < 0.0001, with post hoc Dunnett’s multiple comparison test. **E** One-way ANOVA test, F (6, 50) = 9.996, p < 0.0001, with post hoc Dunnett’s multiple comparison test. **F** One-way ANOVA test, F (6, 50) = 2.061, p = 0.0746, with post hoc Dunnett’s multiple comparison test. **G** One-way ANOVA test, F (6, 50) = 3.461, p = 0.0061, with post hoc Dunnett’s multiple comparison test. All post hoc Dunnett’s multiple comparison tests were performed with the vehicle group as control. All, except the group of (+TNF-α + IL-1α, 100 ng/ml), n = 8 fields of 4 independent dishes from one time differentiations of two control healthy hiPSC lines each, n(+ TNF-α + IL-1α, 100 ng/ml) = 9 fields of 4 independent dishes from one time differentiations of two control healthy hiPSC lines each. *p < 0.05, **p < 0.01, ***p < 0.001, ****p < 0.0001

Next, we added these cytokines to the neuron culture medium to ascertain the direct effect of cytokines on MAP2 + dendrites. Although no significant difference in *TNF-α* expression was observed between ASD-GM-CSF MΦ and TD-GM-CSF MΦ on qRT-PCR analysis after neuron medium culture, we considered it hasty to conclude that one is more important than the other based solely on this qRT-PCR result as TNF-α and IL-1α are known to interact and induce each other’s expression with further additive effects [42]; thus, we used both cytokines in the addition experiment (Fig. 4A). The expression of each receptor in our hiPSC-derived neurons was confirmed by ICC and qRT-PCR (Additional file 1: Fig. S4). Neurons cultured with these cytokines had shorter dendrites (Fig. 4B–E). Furthermore, this effect was stronger when both were added than when only one was added. The number of dendrites was still the same in all groups (Fig. 4F). Still, the results for the number of branches were not as consistent as when co-cultured with macrophages, with no difference between groups, except for the addition of TNF-α at higher concentrations (Fig. 4G). These results confirmed that cytokines affect dendrites.

### Administration of neutralizing antibodies against TNF-α and IL-1α prevented dendritic shortening induced by GM-CSF MΦ

**Fig. 5:**
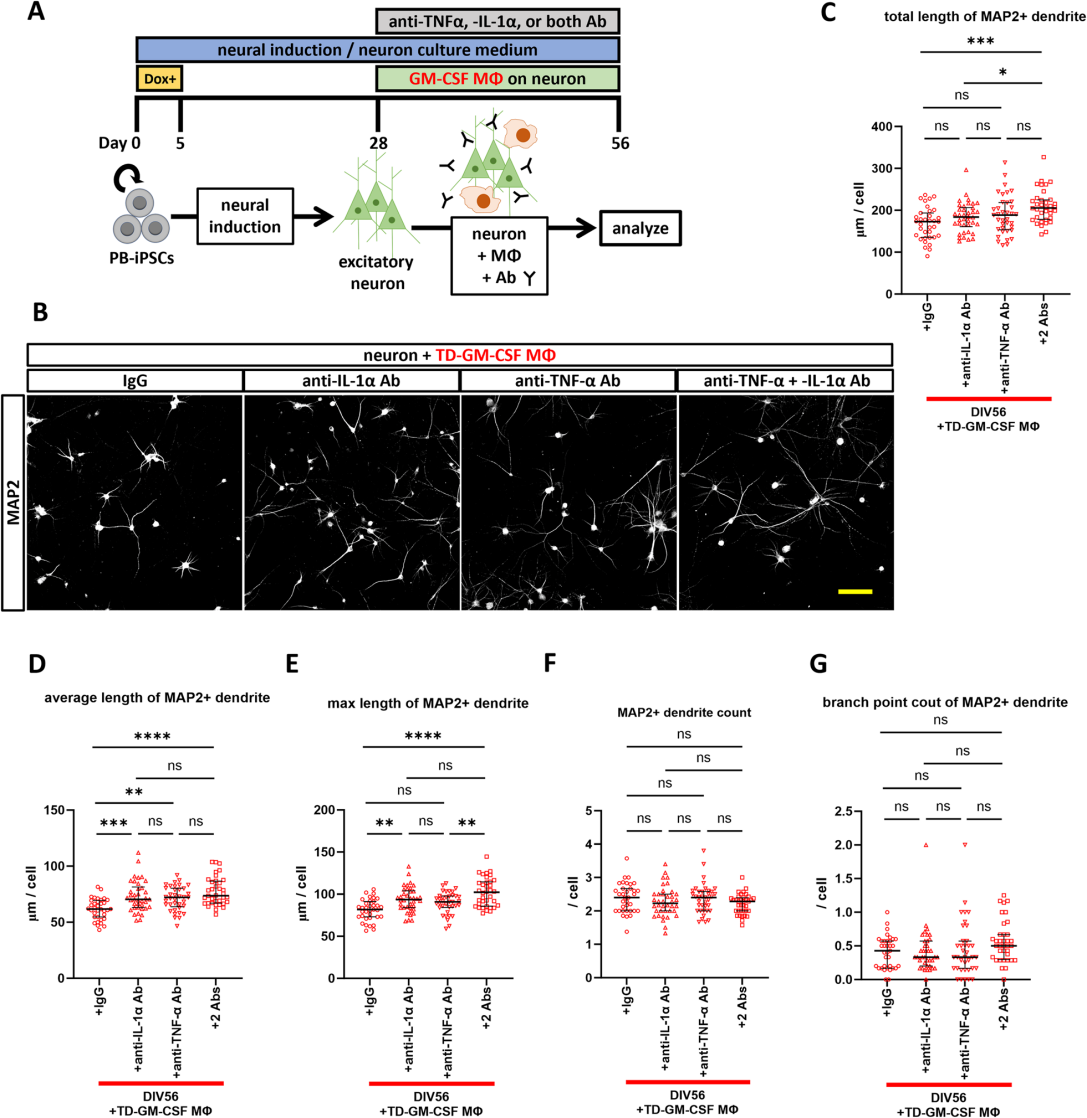
Administration experiment of neutralizing antibodies of TNF-α and IL-1α with TD-GM-CSF MΦ. **A** Summary of the administration experiment of neutralizing antibodies; the same protocol was used with ASD-GM-CSF MΦ. **B** Representative images at DIV56 of immunostaining of MAP2 + dendrite co-cultured with TD-GM-CSF MΦ and simultaneous administration of neutralizing antibodies for TNF-α, IL-1α, or both, or corresponding isotype antibodies (IgG) at a concentration of 1 μg/ml. All neurons were induced from 201B7 hiPSC line, and co-cultured with macrophages of TD4. Scale bar: 100 μm. **C**–**G** Results of (**C**) total length of MAP2 + dendrite, **D** average length of MAP2 + dendrite, **E** max length of MAP2 + dendrite, **F** MAP2 + dendrite count, and **G** branch point count of MAP2 + dendrite. The shortening of MAP2 + dendrites observed in co-culture with TD-GM-CSF MΦ was prevented by the simultaneous administration of neutralizing antibodies. **C**–**F** One-way ANOVA test with post hoc Tukey’s multiple comparison test (**C**) F (3, 142) = 6.606, p = 0.0003, **D** F (3, 142) = 11.25, p < 0.0001, **E** F (3, 142) = 13.05, p < 0.0001, (F) F (3, 142) = 1.544, p = 0.2058. **G** Brown–Forsythe ANOVA test, F (3.00, 127.0) = 1.759, p = 0.1584, with post hoc multiple comparison test of Dunnett’s T3. n(TD-GM-CSF MΦ + IgG) = 35 fields of 15 independent dishes from five times differentiations of two control healthy hiPSC lines each, n(TD-GM-CSF MΦ + anti-IL-1α Ab) = 38 fields of 18 independent dishes from five times differentiations of two control healthy hiPSC lines each, n(TD-GM-CSF MΦ + anti-TNF-α Ab) = 37 fields of 17 independent dishes from five times differentiations of two control healthy hiPSC lines each, and n(TD-GM-CSF MΦ + 2Abs) = 36 fields of 16 independent dishes from five times differentiations of two control healthy hiPSC lines each. *p < 0.05, **p < 0.01, ***p < 0.001, ****p < 0.0001

**Fig. 6:**
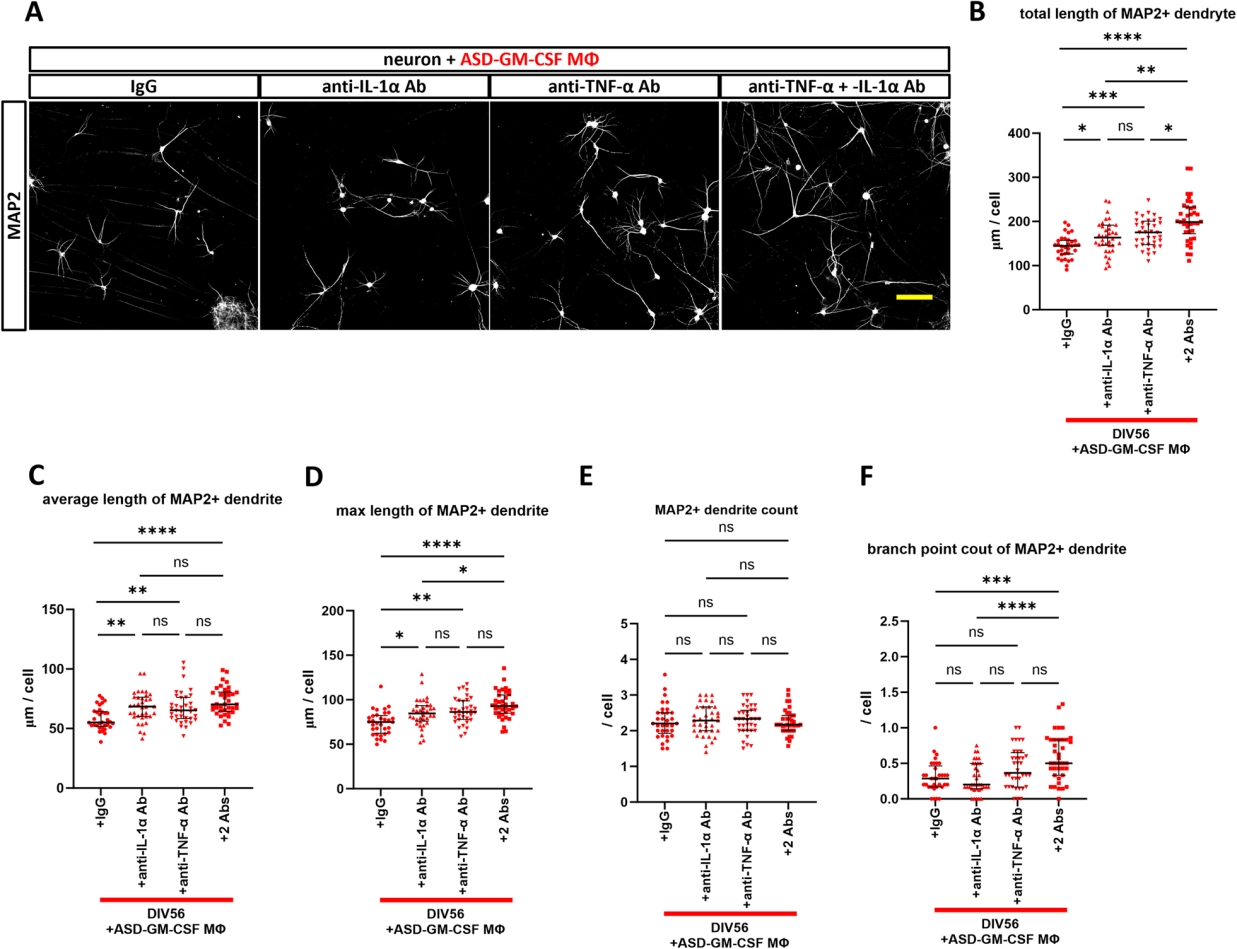
Administration experiment of neutralizing antibodies of TNF-α and IL-1α with ASD-GM-CSF MΦ. **A** Representative images at DIV56 of immunostaining of MAP2 + dendrite co-cultured with ASD-GM-CSF MΦ and simultaneous administration of neutralizing antibodies for TNF-α, IL-1α, or both, or the corresponding isotype antibodies (IgG) at a concentration of 1 μg/ml. All neurons were induced from 201B7 hiPSC line, and co-cultured with macrophages of ASD1 were used in these images. Scale bar: 100 μm. **B**–**F** Results of (**B**) total length of MAP2 + dendrite, **C** average length of MAP2 + dendrite, **D** max length of MAP2 + dendrite, **E** MAP2 + dendrite count, and **F** branch point count of MAP2 + dendrite. The shortening of MAP2 + dendrites observed in co-culture with ASD-GM-CSF MΦ was prevented by the simultaneous administration of neutralizing antibodies. **B** Brown–Forsythe ANOVA test, F (3.00, 120.7) = 16.68, p < 0.0001, with post hoc multiple comparison test of Dunnett’s T3. **C**–**D** One-way ANOVA test with post hoc Tukey’s multiple comparison test (**C**) F (3, 139) = 9.166, p < 0.0001, **D** F (3, 139) = 11.37, p < 0.0001, **E** F (3, 139) = 0.2045, p = 0.8931. **F**) Brown–Forsythe ANOVA test, F (3.00, 127.6) = 10.12, p < 0.0001, with post hoc multiple comparison test of Dunnett’s T3. n(ASD-GM-CSF MΦ + IgG) = 33 fields of 13 independent dishes from five times differentiations of two control healthy hiPSC lines each, n(ASD-GM-CSF MΦ + anti-IL-1α Ab) = 36 fields of 16 independent dishes from five times differentiations of two control healthy hiPSC lines each, n(ASD-GM-CSF MΦ + anti-TNF-α Ab) = 36 fields of 16 independent dishes from five times differentiations of two control healthy hiPSC lines each, and n(ASD-GM-CSF MΦ + 2Abs) = 38 fields of 16 independent dishes from five times differentiations of two control healthy hiPSC lines each. *p < 0.05, **p < 0.01, ***p < 0.001, ****p < 0.0001

To confirm that cytokines released from GM-CSF MΦ induce dendritic shortening and to explore the rescue possibility of neutralizing antibodies, we administered neutralizing antibodies against TNF-α and IL-1α for 28 days in neuron culture medium (Fig. 5A). We conducted preliminary experiments with the TD-GM-CSF MΦ of three individuals at antibody concentrations of 100 ng/ml and 1 μg/ml and obtained no rescue effect at the antibody concentration of 100 ng/ml (Additional file 1: Fig. S5). Simultaneous administration of neutralizing antibodies at the antibody concentration of 1 μg/ml prevented the shortening of MAP2 + dendrites in co-culture with TD-GM-CSF MΦ (Fig. 5B–G) and ASD-GM-CSF MΦ (Fig. 6A–F). The respective neutralizing antibodies exerted a preventive effect to some extent. Still, simultaneous administration of both antibodies was more effective, especially in co-culture with ASD-GM-CSF MΦ, with a significant prevention effect compared to the two antibodies observed in total length and a maximum length of dendrites and branch points (Fig. 6B, D, and F).

## Discussion

In the present study, we showed that human GM-CSF MΦ affect human neurons by inhibiting the outgrowth of MAP2 + dendrites in our co-culture system of hiPSC-derived neurons and macrophages differentiated from peripheral blood monocytes, and that this inhibitory effect is more severe in the GM-CSF MΦ of individuals with ASD than that in TD individuals. In addition, we concluded that two pro-inflammatory cytokines, TNF-α and IL-1α, secreted by GM-CSF MΦ, play a crucial role in this phenomenon. A previous study in a rodent model showed that TNF-α and IL-1α secreted from activated microglia in the mPFC lead to the shortening of dendrite length and reduction of dendrite branches [29]. However, to the best of our knowledge, our results are the first to show this relationship in human cells. In this aforementioned rodent model, microglial activation was induced by repeated social defeat stress, and the neuronal damage caused by secreted cytokines led to social avoidant behavior as a result [29]. Taken together with our results that GM-CSF MΦ of individuals with ASD had more severe effects, a similar mechanism could cause the vulnerability to social stress and a tendency for social withdrawal to be observed in individuals with ASD [30, 31]. Additionally, the hiPSC-derived neurons we used in this study were still in the process of maturation from DIV28 to DIV56, in which period macrophages were co-cultured. Thus, the observed macrophage effects on neurons suggest that immune abnormalities underlie autism pathology from the brain developmental stage and may lead to changes as indicated by increased short-range projection and decreased long-range projection in human imaging study [43]. Reduced MAP2 expression was reported in the prefrontal cortex of two individuals with ASD in postmortem brain case reports [44]. Furthermore, neurons derived from the iPSCs of individuals with 16p11.2 duplication syndrome, a known genetic risk factor for ASD, have shortened dendrites and reduced synaptic density [45]. A previous study analyzing IFN-γ effects on human neurons have shown that IFN-γ led to increased neurite outgrowth in hiPSC-derived neurons and concluded that the phenotype is similar to that observed in hiPSC neurons from individuals with ASD [41]. Although these results seem contradictory, ASD is a heterogeneous disorder [46], and its underlying pathology is unlikely to be homogeneous. Thus, a variety of pathologies may be identified, especially in studies with small sample sizes like our study.

TNF-α is known to modulate brain development and function. Although TNF-α is released from various types of cells, including monocytes, T cells, mast cells, natural killer cells, keratinocytes, fibroblasts, and neurons, it is predominantly released from macrophages and microglia [47, 48]. In the CNS, TNF-α signaling has been shown to exert several essential functions, including in neurogenesis, synaptic plasticity and scaling, injury-mediated activation of microglia and astrocytes, and the regulation of BBB permeability [49–52]; as such, abnormalities in TNF-α signaling can contribute to the onset of neuropsychiatric disorders, including ASD. In fact, the expression of TNF-α in the plasma, cerebrospinal fluid, and postmortem brain samples is higher in individuals with ASD than in TD individuals [53–55]. Our group has also reported elevated TNF-α levels in blood samples, lymphoblastoid cell lines, and macrophages of individuals with ASD [27, 32, 46]. However, only a limited number of studies have directly tested the effects of TNF-α using human neural progenitor cells or neurons, and the results were controversial. In the ischemic model, TNF-α protected neural progenitor cells to survive [56], whereas TNF-α disrupted brain organoid development of schizophrenia patients [57]; the combination treatment of TNF-α with IL-17A led to neurite disturbances in multiple sclerosis patient iPSC-derived neurons, while single cytokine treatment did not [58]. Although background pathologies and TNF-α concentrations vary, our result provided new insight into the detrimental potential of TNF-α, by itself, to inhibit dendritic outgrowth in human neurons. This finding is also supported by the fact that the effect of macrophages on MAP2 + dendrites is parallel to the TNF-α expression ratio of GM-CSF MΦ/M-CSF MΦ evident in our previous study. This included the differences between the TD and ASD.

The IL-1 family constitutes 11 cytokines and ten receptors, the former being divided into three subfamilies [59, 60]. IL-1 is a subfamily of pro-inflammatory cytokines secreted by diverse cells, including immune cells such as macrophages and monocyte [61]. Although IL-1α and IL-1β bind to the same receptor and trigger the same signal transduction, only IL-1α is active in a precursor state and acts as an integral membrane protein, especially in macrophages [60, 62]. IL-1 has been shown to modulate neuronal signaling in homeostasis, such as sleep and memory formation, and in diseases, such as chronic fatigue, depression, anxiety and panic disorder, possibly in a concentration-dependent manner [63]. Most of the studies have analyzed IL-1β, and less is known about IL-1α. Human studies have reported that an association between the genetic mutation of IL-1 receptor accessory protein like 1 with learning disabilities and autism-like syndromes [64], and that IL-1α promotes neurogenesis in adult human mesenchymal stem cells [65]; however, the direct effect of IL-1α on brain cells remains to be elucidated. Our results likewise suggested such a new insight into IL-1α action, showing that it can directly affect neurons and has the potential to alter dendrites. Interestingly, concerning the CNS, it has been reported that IL-1α and IL-1β, in combination with TNF-α secreted from microglia, induce different types of astrocytes, neurotoxic and protective, respectively [42]. This additive effect of microglia-derived TNF-α and IL-1α on astrocytes may cause the neuronal and behavioral changes shown in the rodent model [29]; however, this effect is not expected to exist in our culture model, which does not include astrocytes.

A recent study on amyotrophic lateral sclerosis (ALS) showed the therapeutic value of peripheral macrophages [66]. In this mouse model study, modifying macrophages at the periphery could suppress pro-inflammatory microglial responses, shift microglial activity to protect neuronal survival, and prevent disease progression. In addition, it is known that during certain diseases or injuries, such as ischemic stroke, traumatic brain injury, and neurodegenerative diseases, peripherally derived macrophages may infiltrate the brain where they show an M1-microglia like phenotype [67–69]; however, in this ALS model, peripheral macrophages do not enter the brain. Whether or not this hypothesis applies to ASD remains to be elucidated, but these results indicate the possibility of treating ASD simply by modifying peripheral macrophages with the peripheral administration of neutralizing cytokine antibodies, as partially shown here. Fortunately, some neutralizing antibodies against IL-1α and TNF-α are already in practical use for treating cancers, rheumatoid arthritis, and other chronic local inflammatory diseases [70, 71]. Future research on accumulated evidence from human ASD trials is expected.

### Limitations

One of the limitations of the present study was the small sample size with a gender bias toward males; it is therefore unclear whether the results also apply to a more diverse general population. Second, the condition of the cell culture model was far from the in vivo brain environment in the absence of GABAergic inhibitory neurons and glial cells, especially astrocytes, and macrophages were artificially polarized by GM-CSF or M-CSF to either end of its spectrum of phenotype [72]. Furthermore, it has been shown that these can rapidly switch from one phenotype to another, in vivo [73, 74]. It has not been directly analyzed whether the high expression of inflammatory cytokines in GM-CSF MΦ and the differences between TD and ASD were maintained under co-culture conditions. Additionally, neurons were derived from two healthy control subjects; thus, we could not analyze the interaction effect of neurons and macrophages in individuals with ASD. However, using such simplified culture systems, we could identify macrophage abnormalities in ASD.

## Conclusions

In conclusion, our co-culture system revealed the adverse effects of the GM-CSF MΦ of individuals with ASD on neuronal dendrites via the secretion of the pro-inflammatory cytokines, IL-1α and TNF-α. These results will be useful for understanding the pathobiology of ASD and for future drug discovery.

### Supplementary Information

**Additional file 1**. Supplementary methods and figures.

## Data Availability

The datasets used and/or analyzed during the current study are available from the corresponding author on reasonable request.

## Abbreviations

ASD: Autism spectrum disorder
CNS: Central nervous system
PBMCs: Peripheral blood mononuclear cells
BBB: Blood–brain barrier
IFN-γ: Interferon-γ
IL: Interleukin
CAMs: CNS-associated macrophages
TNF-α: Tumor necrosis factor-α
GM-CSF MΦ: Granulocyte–macrophage colony-stimulating factor-induced macrophages
M-CSF MΦ: Macrophage colony-stimulating factor-induced macrophages
TD: Typically developed
mPFC: Medial prefrontal cortex
hiPSC: Human iPS cell
ADOS-2: Autism Diagnostic Observation Schedule-2
AQ-J: Autism Quotient-Japanese version
FIQ: Full intelligence quotient
qRT-PCR: Quantitative reverse transcription-polymerase chain reaction
DIV: Day in vitro
ICC: Immunocytochemistry
PBS: Phosphate-buffered saline
MAP2: Microtubule-associated protein 2
ANOVA: Analysis of variance
HSD: Honest significant test
Tuj-1: Beta III tubulin
vGlut2: Vesicular glutamate transporter 2
IFN-γ: Interferon-γ
ALS: Amyotrophic lateral sclerosis
