# Supplementary material for: Granulocyte macrophage colony-stimulating factor-induced macrophages of individuals with autism spectrum disorder adversely affect neuronal dendrites through the secretion of pro-inflammatory cytokines
Source: Mol Autism. 2024 Feb 21;15:10. doi: 10.1186/s13229-024-00589-2 (PMC10882766; doi:10.1186/s13229-024-00589-2)

**Supplemental Information**

**Supplementary Methods**

**Quantitative reverse transcription-PCR**

Total RNA was extracted from cultured cells using the AllPrep DNA/RNA/Protein Mini Kit (Qiagen, Hilden, Germany), according to the manufacturer’s protocol. RNA concentration was determined by measuring absorbance at 260 nm. First-strand cDNA was synthesized from total RNA using an iScript kit (Bio-Rad Laboratories, Hercules, CA, USA), and quantitative RT-PCR was performed using SYBR Premix Ex *Taq*II (Tli RNaseH Plus, Takara Bio Inc., Shiga, Japan) with a QuantStudio 6 real-time PCR system (Applied Biosystems, Thermo Fisher Scientific Inc.). Relative quantification of the expression levels of target genes was performed following the delta CT method, using two constitutively expressed genes as internal controls: *β-actin* (*ACTB*) and *Cyclophilin A* (*CyA*). Primer sequences were as follows: *β-actin*, forward 5´- GATGTGGATCAGCAAGCA-3´, reverse 5´-AGAAAGGGTGTAACGCAACTA-3´; *CyA*, forward 5´-GCAGACAAGGTCCCAAAG-3´, reverse 5´-GAAGTCACCACCCTGACAC-3´; *IL-1α*, forward 5´-TGTATGTGACTGCCCAAGATGAAG-3´, reverse 5´-AGAGGAGGTTGGTCTCACTACC-3´; *IL-10*, forward 5´-GCCTAACATGCTTCGAGATC-3´, reverse 5´-TGATGTCTGGGTCTTGGTTC-3´;　 *IFN-γ*, forward 5´-TCGGTAACTGACTTGAATGTCCA-3´, reverse 5´-TCCTTTTTCGCTTCCCTGTTTT-3´; *TNF-α*, forward 5´-GGCAGTCAGATCATCTTCTCG-3´, reverse 5´-CAGCTTGAGGGTTTGCTACA-3´;　*IL-1R1*, forward 5´-GTGCTTTGGTACAGGGATTCCTG-3´, reverse 5´-CACAGTCAGAGGTAGACCCTTC-3´; *TNFR1*, forward 5´-CGCTACCAACGGTGGAAGTC-3´, reverse 5´-CAAGCTCCCCCTCTTTTTCA-3´. The qRT-PCR data are presented as the mean ± SEM. Comparisons were performed using unpaired t-test or Welch’s t-test.

**Electrophysiological analysis**

For electrophysiological experiments, hiPSC-derived neurons cultured on coverslips were transferred to a recording chamber, submerged in the culture solution as mentioned in the methods, and maintained during recording with the room air at 25°C. Cells were visualized using an upright microscope (BX51WI-35FL; Olympus, Tokyo, Japan) equipped with infrared illumination and differential interference contrast videomicroscopy capabilities. The neurons were voltage- or current-clamped in the conventional whole-cell configuration, using a Multiclamp 700A amplifier (Axon Instruments). Patch pipettes were pulled from borosilicate glass and filled with an intracellular solution (141 mM K-gluconate, 4 mM KCl, 2 mM MgCl2, 2 mM Mg-ATP, 0.3 mM Na2-GTP, 0.2 mM EGTA, and 10 mM HEPES; pH 7.25 with KOH). The calculated Cl^-^ equilibrium potential was -74 mV at 32°C. Data acquisition and stimulation were controlled using Signal 4 software with a Power 1401 interface equipment (Cambridge Electronic Design).

**Voltage clamp recording**

For the voltage-clamp recording, the capacitance of the pipette was compensated, while the series resistance was not compensated and was continuously monitored. Recordings with a stable series resistance of < 20 MΩ were analyzed. Current signals were low-pass filtered at 600 Hz and digitized at a sampling frequency of 10 kHz. To record EPSCs, cultured neurons were held at -70 mV. In our experimental condition, GABAA receptor-mediated postsynaptic currents were outward and too small to be detected in most events, whereas EPSCs were detected as definite inward currents. Meanwhile, sEPSCs were recorded in the artificial cerebrospinal fluid with no GABAA receptor antagonists to maintain both excitatory and inhibitory neuronal activity.

**Current clamp recording**

In the current-clamp recordings, series resistance was monitored and canceled using a bridge circuit, and the capacitance of the pipette was compensated. Voltage signals were low-pass filtered at 10 kHz and digitized at 20 kHz. The baseline membrane potential was maintained near -70 mV with the current injection. To examine the action potential and subthreshold membrane properties, we recorded membrane potential responses to hyperpolarizing current pulses (500 ms in duration).

**Supplementary Figures**

**Supplementary Fig. S1. Immunocytochemistry of hiPS cells and hiPSC-derived neurons, and electrophysiological analysis of hiPSC-derived neurons.**

(A) A scheme of MAP2-positive dendrite tracing. The red line indicates the length of the dendrite, the yellow arrowhead indicates the number of dendrites, and the green arrowhead indicates the branch point. In this cell, each parameter is calculated as follows: total length (sum of (i) to (iv)), average length (sum of (i) to (iv) / 4), max length (length of (i)), dendrite count (3), and branch point (1). (B) Expression of pluripotent stem cell markers in two hiPS cell lines using immunocytochemistry. Scale bar: 200 μm. Images were randomly acquired using a KEYENCE BZ-X810 microscope with BZ-X800 Viewer acquisition software (KEYENCE, Osaka, Japan). (C) Immunocytochemistry of hiPSC-derived neurons at DIV56. The expression of beta III tubulin (Tuj-1) was observed, but that of glial fibrillary acidic protein (GFAP) was absent. Scale bar: 100 μm. (D) Electrophysiological analysis of hiPSC-derived neurons. Representative results of current clamp recordings (middle) and voltage clamp recordings (lower) were shown. Scale bar (upper picture): 50 μm. (E) Gene expression of *IL-1α* and *IL-10* to characterize GM-CSF MΦ and M-CSF MΦ, and *IFN-γ* on day6 in macrophage differentiation medium.


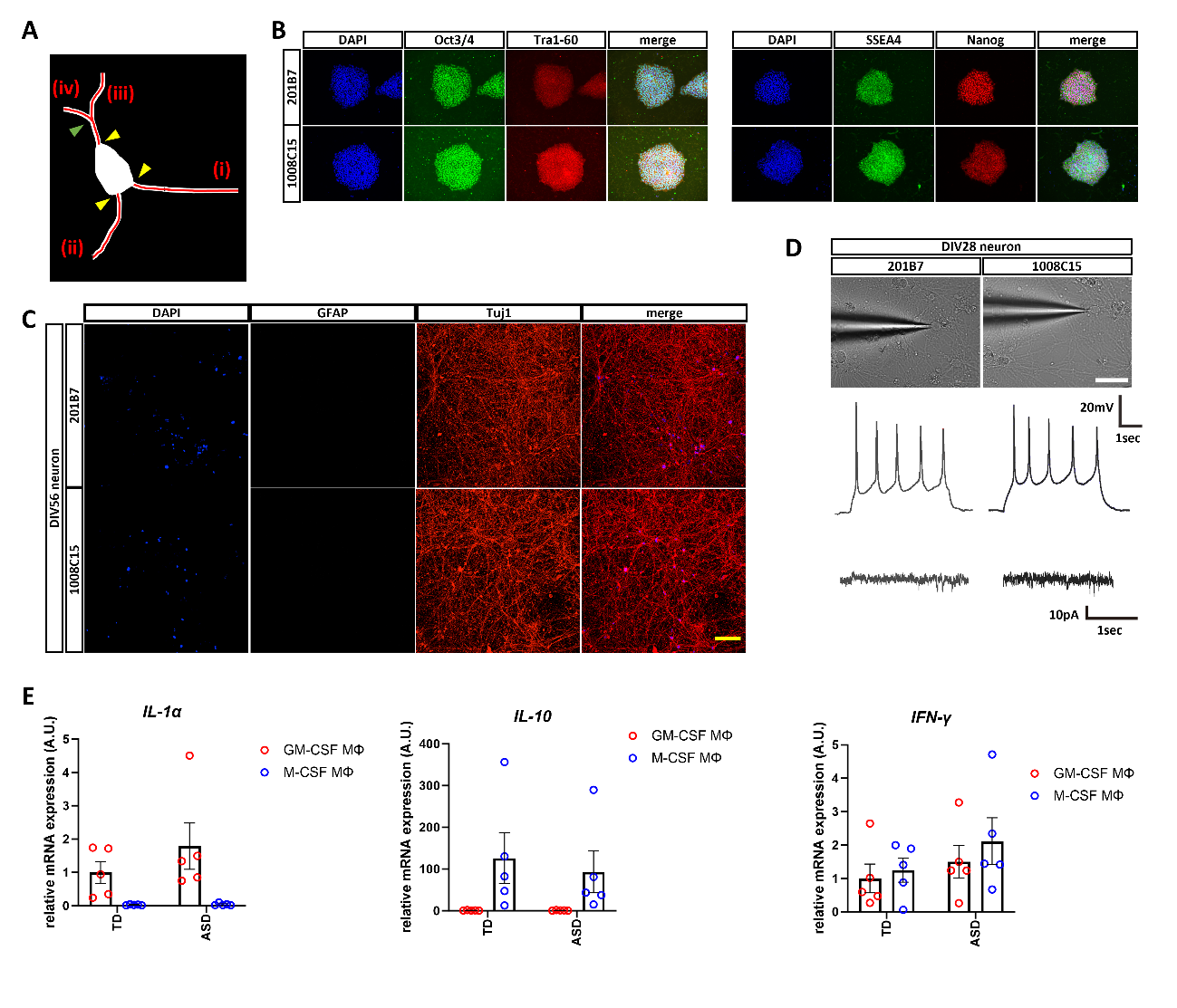


**Supplementary Fig. S2. M-CSF MΦ had no impact on neuronal dendrites.**

(A) Immunocytochemistry of hiPSCs-derived glutamatergic neurons induced from 1008C15 hiPSC line at DIV28 or DIV56, with or without GM-CSF MΦ of TD5 or ASD5. Scale bar: 100 μm. (B) The number of MAP2 positive cells at DIV56. One-way ANOVA test, F (2, 105) = 1.346, p = 0.2648, with posthoc Tukey’s multiple comparison test. n(DIV56-neuron)= 31 fields of 14 independent dishes from five times (201B7) and 4 times (1008C15) differentiations of two control healthy hiPSC lines each, n(TD-GM-CSF MΦ)= 39 fields of 19 independent dishes from five times differentiations of two control healthy hiPSC lines each, and n(ASD-GM-CSF MΦ)= 38 fields of 18 independent dishes from five times differentiations of two control healthy hiPSC lines each. (C) Representative images of immunostaining of MAP2+ dendrites. All neurons were induced from the 201B7 hiPSC line, and co-cultured with macrophages of TD2 or ASD1 were used in these images. Scale bar: 100 μm. (B-F) Results of (D) total length of MAP2+ dendrite, (E) average length of MAP2+ dendrite, (F) max length of MAP2+ dendrite, (G) MAP2+ dendrite count, and (H) branch point count of MAP2+ dendrite from 3 participants in each group. Dendrites of co-cultured neurons with ASD-M-CSF MΦ showed no significant change compared to TD-M-CSF MΦ at DIV56. (D) Unpaired t test, t = 1.482, p = 0.1453. (E) Unpaired t test, t = 0.3228, p = 0.7484. (F) Unpaired t test, t = 0.1706, p = 0.8653. (G) Unpaired t test, t = 0.9360, p = 0.3542. (H) Unpaired t test, t = 1.184, p = 0.2428. n(TD-M-CSF MΦ)= 24 fields of 12 independent dishes from three times differentiations of two control healthy hiPSC lines each, and n(ASD-M-CSF MΦ)= 23 fields of 11 independent dishes from three times differentiations of two control healthy hiPSC lines each.


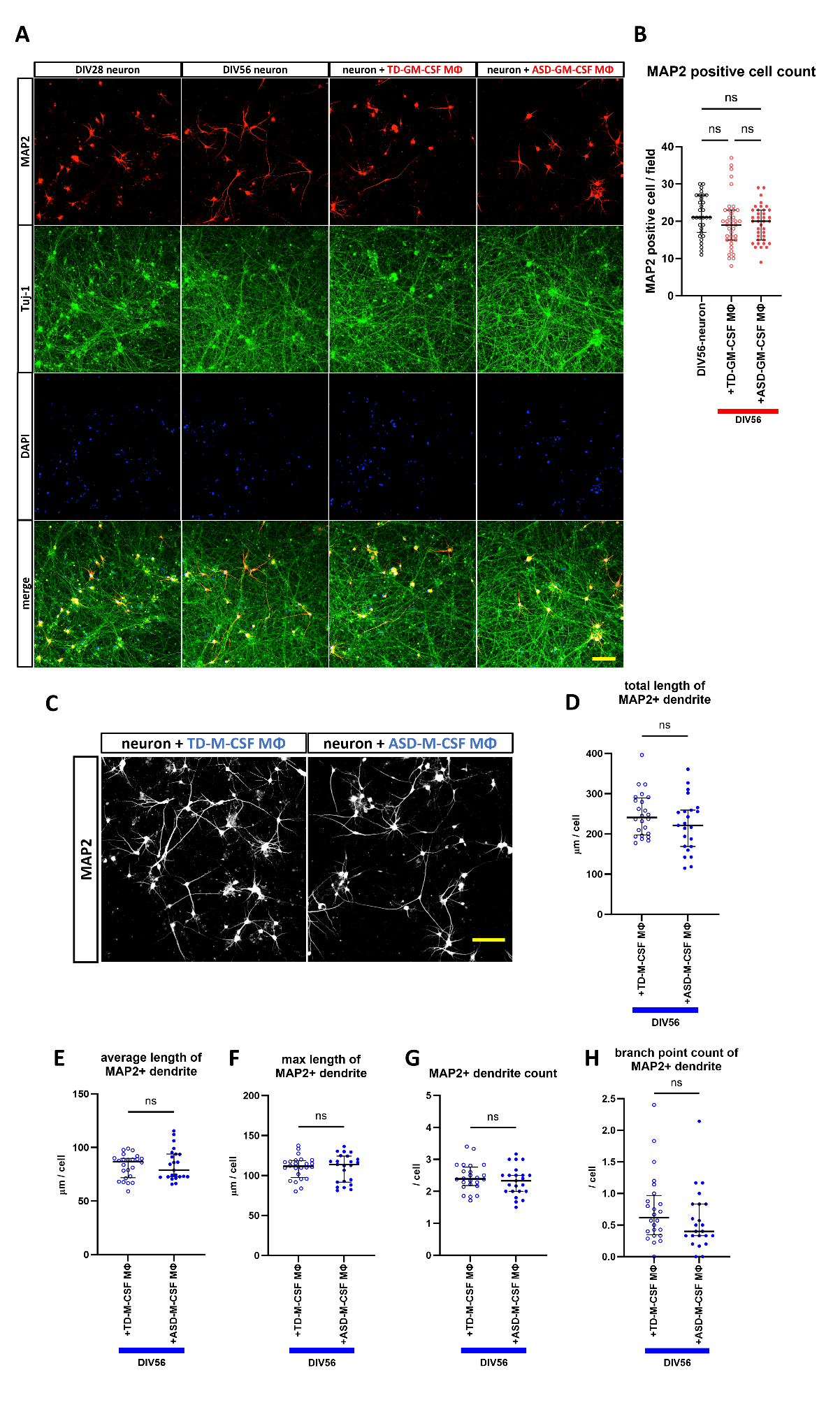


**Supplementary Fig. S3. ASD-GM-CSF MΦ have higher expression of pro-inflammatory cytokines compared to TD-GM-CSF MΦ.**

(A) Summary of the qRT-PCR analysis of macrophage cultured in neuronal medium. (B) Gene expression of *IL-1α* was predominantly higher in ASD-GM-CSF MΦ compared to TD-GM-CSF MΦ but no difference was observed in *TNF-α* (Welch’s t test, t = 1.10, p = 0.32; *TNF-α*, and t = 3.07, p = 0.03; *IL-1α*).


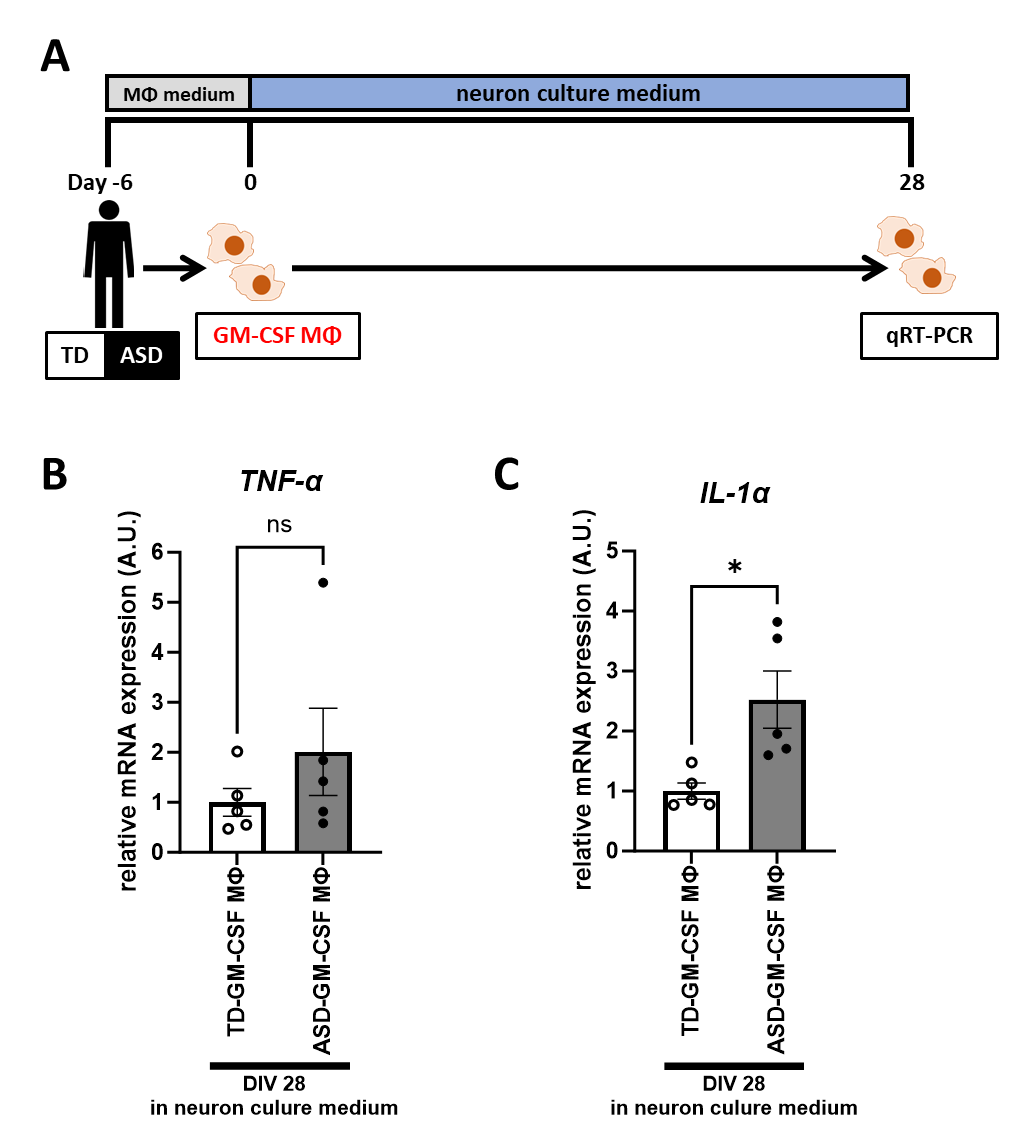


**Supplementary Fig. S4. Expression of cytokine receptors in hiPSC-derived neurons.**

Immunocytochemistry of (A) IL-1 receptor 1 and (C) TNF receptor 1, and gene expression of (B) *IL-1R1* and (D) *TNFR1* of hiPSCs-derived neurons at DIV28. Scale bar: 30 μm. No difference was observed between two hiPS cell lines (unpaired t-test, t=0.612, p=0.558; *IL-1R1,* and t=1.11, p=0.298; *TNFR1*).


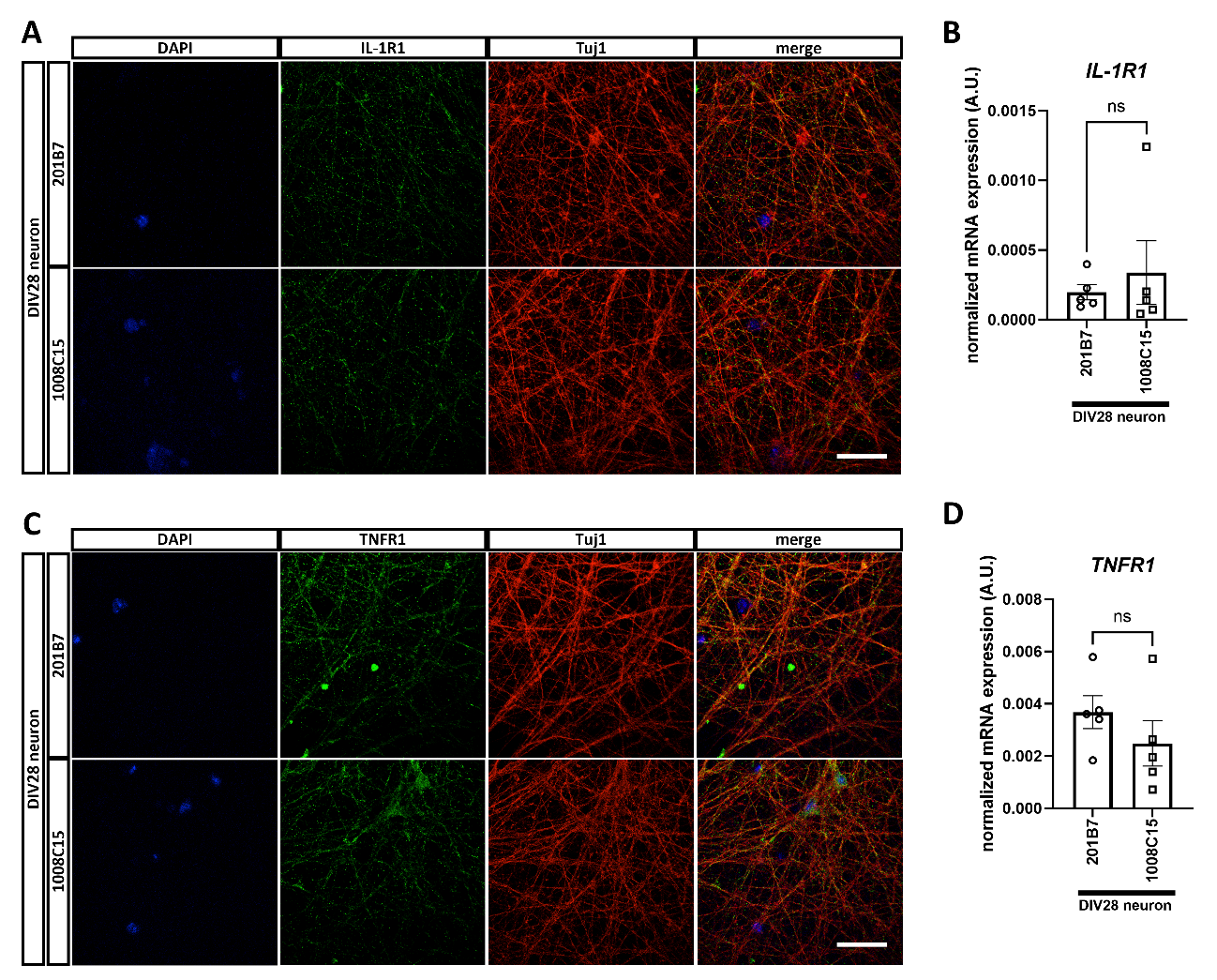


**Supplementary Fig. 5. Administration experiment of neutralizing antibodies of TNF-α and IL-1α with TD-GM-CSF MΦ.**

The administration experiment of neutralizing antibodies; neurons were co-cultured with TD-GM-CSF MΦ from three participants and simultaneous administration of neutralizing antibodies for TNF-α and IL-1α or corresponding isotype antibodies (IgG) at a concentration of 100 ng/ml or 1 μg/ml. (A-E) Results of (A) total length of MAP2+ dendrite, (B) average length of MAP2+ dendrite, (C) max length of MAP2+ dendrite, (D) MAP2+ dendrite count, and (E) branch point count of MAP2+ dendrite. (A) One-way ANOVA test, F (2, 58) = 10.50, p = 0.0001, with posthoc Tukey’s multiple comparison test. (B) One-way ANOVA test, F (2, 58) = 13.50, p < 0.0001, with posthoc Tukey’s multiple comparison test. (C) One-way ANOVA test, F (2, 58) = 17.59, p < 0.0001, with posthoc Tukey’s multiple comparison test. (D) One-way ANOVA test, F (2, 58) = 1.92, p = 0.156, with posthoc Tukey’s multiple comparison test. (E) One-way ANOVA test, F (2, 58) = 6.06, p = 0.0041, with posthoc Tukey’s multiple comparison test. n(TD-GM-CSF MΦ +IgG)= 21 fields of seven independent dishes from three times differentiations of two control healthy hiPSC lines each, n(TD-GM-CSF MΦ +2Abs (100 ng/ml))= 20 fields of seven independent dishes from five times differentiations of two control healthy hiPSC lines each, and n(TD-GM-CSF MΦ +2Abs (1 μg/ml))= 20 fields of seven independent dishes from five times differentiations of two control healthy hiPSC lines each. * p < 0.05, ** p < 0.01, *** p < 0.001, **** p < 0.0001


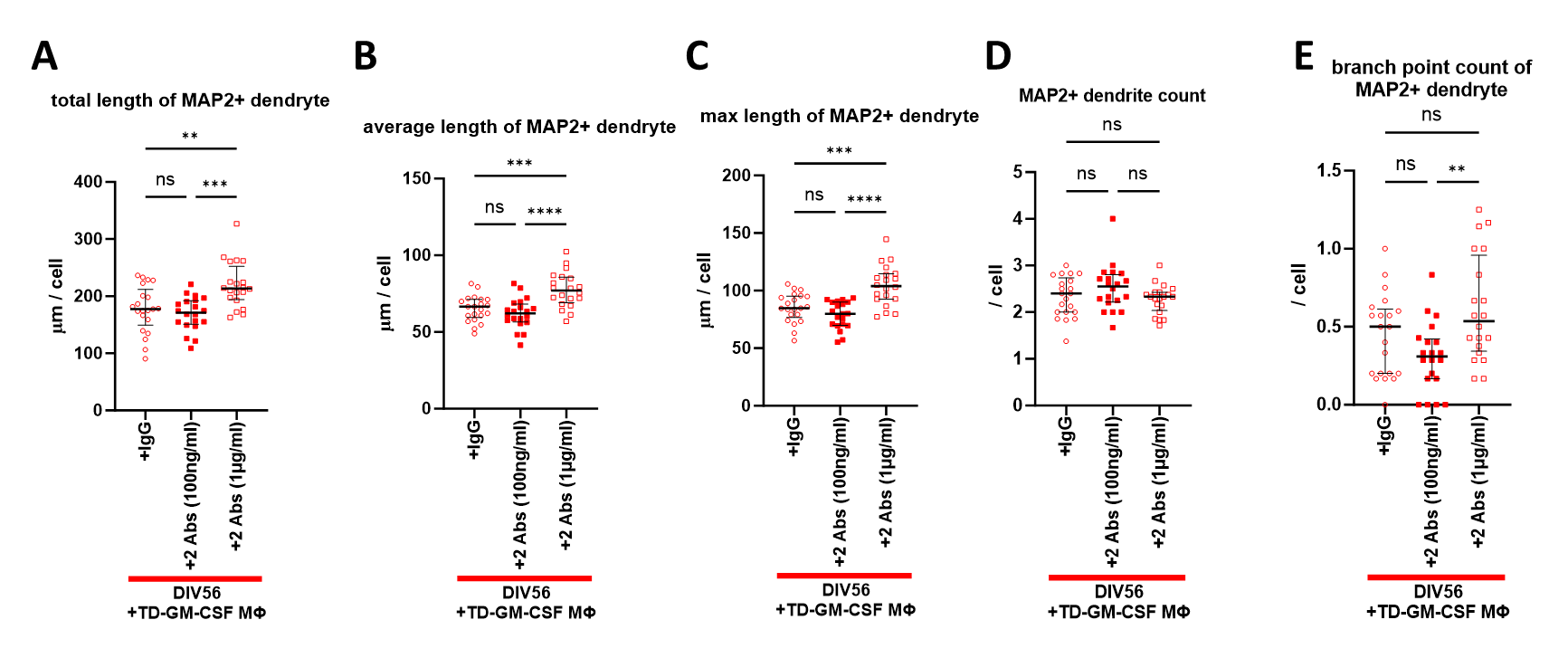

Supplement: Supplementary file 1 — Additional file 1. Supplementary methods and figures. [file 13229_2024_589_MOESM1_ESM.docx]
